# The Testamentary Habits of Legators

**Published:** 1921-07-30

**Authors:** 


### LEGACIES AND THE HOSPITAL BUDGET.

### The Testamentary Habits of Legators.

FROM one of the appendices of the report of Lord Cave's Committee, it is shown that last year £266,000 was received in free legacies by 113 of the London hospitals, or rather more than 10 per cent. of their total ordinary income. In the provinces the sum received by 452 hospitals was £269,000 out of a revenue of £3,721,000, and in Scotland, where the year in question, 1920, was an unusually good one in respect of legacies, £235,000 was received by 107 hospitals, or getting on for a quarter of the total revenue—namely, £1,065,000. Other figures at our disposal show that, so far as London was concerned, the revenue from free legacies was rather under the average, for on the basis of a recent run of ten years the annual amount to be expected is just under £300,000.

There has always been a difference of opinion as to whether all legacies should not be treated as capital, and, in fact, in the rules of quite a number of institutions they are looked upon in this light, and an endeavour is made to fund them each year. This endeavour, however, does not as a rule achieve its object, because what goes into capital on one side has to come out again on the other to meet expenses, and the result is largely the same as if the funds in question were passed through the current account. One of the chief reasons put forward for the funding of legacies is that they may be considered to have come from persons who have been supporters in their lifetime, who now wish to institute a fund from which their support will be continued. This, however, is not the case, as can be easily proved by an inspection of the legacy register of any hospital.

### AN ANALYSIS OF THE LEGACY REGISTER.

Through the courtesy of a secretary, we have recently been able to obtain some statistics which, although they cannot be claimed to be conclusive, are suggestive. In this particular case eighty-eight bequests matured between January 1, 1909, and June 1, 1921. After careful search fifty-three of the persons who thus benefited the hospital were not found in any of the records. The other thirty-five included two ex-patients, one member of the Board of Management, one chaplain, and one president. The rest were persons who had given either annually or from time to time. It was found in most cases that the amount of the legacies had no relation to the amount of the annual or occasional support, or, in other words, that there was no implied intention on the part of the benefactors to perpetuate the periodical amounts given by them during life. It may be of interest to note that of these eighty-eight persons, twenty-eight died within one year of making their wills, and eleven of these within six months. Twenty others died within three years of the bequest, and but a small proportion after ten years. Possibly, taking this fact for what it is worth, the indication, if anything, is that the hospitals benefit most by persons who have not had an opportunity to alter their wills.

Certainly hospital administrators will be in agreement with the statement that, generally speaking, an attempt to discover the best means of securing legacies is most baffling, but no doubt the general basis of policy should be publicity. This we can see from the fact that St. Dunstan's Hostel for the Blind, which has only been in existence for seven years, is frequently mentioned in testamentary directions. How is this publicity to be secured? Advertising in the ordinary way is expensive, and the sum which would, proceeding on commercial practice, be sufficient to bring good results, would be such a heavy proportion of the expenses of a hospital that it would secure the critical attention of the Central Funds and of the larger subscribers, who would not approve of so great a part of their benefactions being spent in a speculative way. But there are many means of advertising; no doubt the hospitals with medical schools are fortunately placed with regard to publicity. Every year they turn out an addition to the already large number of practitioners who always feel kindly towards the place where they received their education. These gentlemen are especially able to help in the matter of legacies, as the doctor, and particularly the general practitioner, is often the family friend, and consulted in matters besides medicine. A patient will sometimes consider that mere money will not fully repay what he has received from his medical adviser, and it is often the case that donations are received by hospitals through members of the medical staff. It is surely equally likely that legacies are influenced in the same way.

### A DISAPPOINTING LEGACY.

If the ways of testators are strange, the ways of subscribers and donors are equally so, and, as we have seen that to quite an appreciable extent the hospital must rely upon its living supporters for future bequests, their wishes must be always studied carefully, or, in other words, it is the business of the hospital to keep upon the best of terms with them. And the wishes of benefactors are various; one will think that it is waste of time and money for a letter of thanks to be written, and will desire but a bare receipt sent at the outlay of a minimum sum of postage. Another will ask that the contribution shall be acknowledged in the personal column of one of the newspapers, this at a cost of several shillings. A third will require that his gift shall be entered in the annual report as anonymous, will probably forget the directions he has given and complain when he fails to find his name in the list with other subscribers. At a well-known hospital there was a lady subscriber who lived in the neighbourhood, and she always desired that the secretary should call upon her on a certain day and at a certain hour to receive her subscription of £5 5s. These conditions were carefully entered in the subscription register, and for many years the secretary did not fail to keep the appointment. One year, however, as he was setting forth he met an important member of the medical staff and remained talking to him for about ten minutes, accordingly arriving at the subscriber's house behind time. He saw the lady and received the usual gift. She said nothing to him about his unpunctuality, but in the following year she died and her sole legacy to the hospital was a grandfather clock, which she stated in the will would possibly be useful to tell the secretary the time. The moral of this story is that as a matter of duty to the institution they serve the chief administrator and his assistants should be most punctilious in their observation of every wish expressed by a supporter.

It is to be remarked that many of the specific legacies received by hospitals are, by direction of the legator, free from legacy duty. Of course, where an institution is left the whole or part of the residue of an estate it pays a share of the estate duty as well. The recommendation of Lord Cave's Committee with regard to the remission of legacy and succession duties in the case of bequests to hospitals will be welcomed by those responsible for obtaining their funds. Of course, institutions sharing in demises of portions of the residue will pay their share of the estate duty, but this, except on very large estates, is not a very weighty matter. If Parliament agrees in this matter of remission of duty the example of the United States will be followed, where there is an exemption from legacy duty and a claim has been made that this has had a marked effect in encouraging bequests. As the report says, there appears to be no reason why the State should intercept a tenth of all sums bequeathed to the sick.

